# A Meta-Analysis of the Incidence of Adverse Reactions of Statins in Various Diseases

**DOI:** 10.1155/cdr/6684099

**Published:** 2025-06-10

**Authors:** Wanying Li, Ding Wang, Caiyue Lin, Tongze Cai, Mei Zhao, Liuguan Liang, Xingxing Zhao, Xin He, Xiaoyue Liang, Jinghui Zheng

**Affiliations:** ^1^Graduate School, Guangxi University of Chinese Medicine, Nanning, Guangxi, China; ^2^Department of Cardiology, The First People's Hospital of Nanning, Nanning, Guangxi, China; ^3^Department of Oncology, Guangxi International Zhuang Medicine Hospital Affiliated to Guangxi University of Chinese Medicine, Nanning, Guangxi, China; ^4^Department of Pulmonary and Critical Care Medicine, The First Affiliated Hospital of Guangxi University of Chinese Medicine, Nanning, Guangxi, China; ^5^Academic Affairs Office, Guangxi University of Chinese Medicine, Nanning, Guangxi, China

**Keywords:** adverse reactions, coronary heart disease, hypercholesterolemia, myalgia, statins

## Abstract

**Introduction:** In clinical practice, patients often avoid or cease statin use due to adverse reactions or noncompliance. To elucidate statin adverse reactions, their variability across diseases, and the factors influencing them, we conducted a high-quality clinical trial–based meta-analysis.

**Materials and Methods:** Clinical randomized controlled trials involving statins and detailed recording of adverse reactions in the following three databases: PubMed, Embase, and Cochrane Library were included. The retrieval was completed by January 31, 2024. All studies will use the ROB2 scale for bias risk assessment.

**Results:** We had included a total of 41 studies, involving a collective sample size of 64,728 individuals. In patients with hyperlipidemia, there was no difference in the overall incidence of total adverse events among four types of statins (*p* = 0.37). Simvastatin 40 mg had fewer statin-related adverse reactions. High-dose statin users experienced no remarkable transaminase elevation 0.00201 (95% CI [0.00004, 0.00398], *I*^2^ = 33%). Creatine phosphokinase (CK) elevation under three times the upper limit was rare with a rate of 0.0043 (95% CI [0.0011, 0.0075], *I*^2^ = 27%). Myalgia rates were comparable between high- and moderate-dose statins (*p* = 0.23). Gastrointestinal symptoms were infrequent with a rate of about 0.02 (95% CI [0.00, 0.01], *I*^2^ = 52%). For patients with coronary heart disease, pravastatin 40 mg resulted in fewer transaminase elevations (*p* < 0.01). There is no difference in myalgia rates between moderate- and high-dose statins (*p* = 0.78). The proportion of myopathy was higher with simvastatin 80 mg compared to other statins. The risk of rhabdomyolysis was dose-dependent (*p* < 0.01). For heart failure patients, elderly patients showed varying risks of CK elevation, gastrointestinal symptoms, and muscle symptoms (*I*^2^ = 71%, 99%, and 99%, respectively). For patients with acute coronary syndrome or acute stroke, the rates of transaminase elevation were higher with simvastatin 40 mg and atorvastatin 80 mg compared to other statins (*p* < 0.01). There is no difference in myalgia rates between rosuvastatin 20 mg and atorvastatin 80 mg (*p* = 0.20). However, the rate of myalgia with atorvastatin 80 mg was higher than that of rosuvastatin 10 mg and atorvastatin 20 mg (*p* < 0.01). For diabetic patients, there was no difference in the effect on transaminases among four statin medications: rosuvastatin 10 and 40 mg, simvastatin 40 mg, and atorvastatin 80 mg (0.00058, 95% CI [0.00000, 0.00464], *I*^2^ = 0%). Additionally, there was no difference in the rates of myalgia among atorvastatin 10, 40, and 80 mg and rosuvastatin 20 and 40 mg (*p* = 0.05).

**Conclusion:** Statins' adverse reactions differ across populations. For those with hypercholesterolemia and diabetes, statins' impact on transaminase levels is similar. Yet patients with coronary heart disease, acute coronary syndrome, or acute stroke show varying responses. Notably, myalgia risk in hypercholesterolemia and coronary disease patients using different statins is comparable, but those with acute coronary syndrome or stroke, especially on high-dose rosuvastatin, have a higher myalgia risk.

## 1. Introduction

From 1990 to 2022, global cardiovascular disease mortality dropped by 34.9%. Despite this progress, ischemic heart disease continues to lead in global disability rates at 2275.9 per 100,000, followed closely by cerebral hemorrhage and ischemic stroke. The prevalence of age-standardized cardiovascular diseases varies significantly by region, ranging from 5881.0 per 10,000 in South Asia to 11,342.6 per 10,000 in Central Asia [1].

A key contributor to the burden of cardiovascular disease is abnormal blood lipids, particularly high levels of low-density lipoprotein cholesterol (LDL-C). Elevated LDL-C accumulates in arterial walls, activating endothelial cells and triggering monocyte attraction into the subendothelial space. This recruitment promotes macrophage activation and inflammation within the intima, leading to atherosclerotic lesion formation, which can result in ischemic heart disease, ischemic stroke, and other manifestations of atherosclerotic cardiovascular disease (ASCVD) [2].

Between 1980 and 2018, global age-standardized levels of nonhigh-density lipoprotein cholesterol (non-HDL-C) notably increased. In men, levels rose from 2.82 to 3.04 mmol/L, while in women, they increased from 2.83 to 3.08 mmol/L. Specifically, in China, age-standardized non-HDL-C levels for men jumped by 0.61 mmol/L, positioning them at 99th globally and surpassing those of several Western countries [3]. As such, managing dyslipidemia and reducing non-HDL-C levels remain crucial strategies in combating cardiovascular and cerebrovascular diseases.

Statins have been a cornerstone in the treatment of high LDL-C, functioning by inhibiting hydroxymethylglutaryl-CoA (HMG-CoA) reductase. These medications can significantly lower LDL cholesterol by 20%–50%, reduce triglyceride levels by 10%–20%, and may slightly increase HDL cholesterol levels [4]. Statins, a treatment modality for nearly 40 years, have proven to be effective and safe. However, some patients may exhibit higher risks of adverse effects, such as myopathy and rhabdomyolysis, particularly with cerivastatin. Thus, balancing efficacy and risk is crucial for protecting patient health [5].

In an examination of adverse event reports (AERs) from the Food and Drug Administration (FDA), it was found that pravastatin, simvastatin, atorvastatin, and rosuvastatin had high incidences of myalgia, rhabdomyolysis, and elevated creatine phosphokinase levels. Notably, rosuvastatin was particularly linked to myalgia, while simvastatin and rosuvastatin showed stronger associations with rhabdomyolysis and increases in creatine phosphokinase [6].

To better understand the real-world implications of statin therapy, a meta-analysis was conducted to assess how the side effects of statins vary across different diseases and to gauge the frequency of various reactions. This information can serve as a valuable resource for physicians in making informed clinical decisions.

## 2. Materials and Methods

This systematic review was conducted in adherence to the PRISMA guidelines and documented using the PRISMA checklist [7]. The study has been prospectively registered in PROSPERO under the registration number CRD42024553987. Given its nature as a meta-analysis, it was exempt from obtaining approval from an institutional review board and patient consent.

### 2.1. Literature Selection

We conducted a comprehensive search for clinical randomized controlled trials (RCTs) involving statin medications, with detailed documentation of adverse reactions, in the PubMed, Embase, and Cochrane Library databases until January 31, 2024. The search terms utilized for this study included keywords related to statins (“statin,” “rosuvastatin,” “atorvastatin,” “pitavastatin,” “lovastatin,” “fluvastatin,” “pravastatin,” and “simvastatin”), randomized clinical trials (“clinical trial,” “clinical study,” and “randomized clinical trial”), and adverse reactions (“side effects,” “adverse effects,” “adverse reactions,” “adverse events,” and “adverse drug reactions”). The complete search strategies for each individual database can be found in Supporting Information 1: Text S1.

### 2.2. Selection Criteria

Studies were included if they met the following inclusion criteria: (1) RCTs, (2) statin comparisons (vs. placebos and other drugs), (3) detailed patient information, (4) clear statin names/doses, (5) follow-up period of at least 1 month, and (6) > 30 participants per group. The exclusion criteria were as follows: (1) unclear reaction details, (2) combined statin types/doses without separate analysis, and (3) missing criteria like elevated transaminase or creatine kinase levels.

### 2.3. Data Extraction and Risks of Bias Assessment

Three researchers (LWY, WD, and LCY) independently filtered and reviewed retrieved trials, resolving disagreements. Two researchers (LWY and WD) then independently extracted data on authors, publication year, location, population, sample size, demographics, medical history, medications, intervention (statin type, dose, and duration), and adverse reactions. Discrepancies were checked by additional authors.

The biased risk assessment used the Cochrane Collaboration's RoB2 tool, featuring five domains: randomization, intervention adherence, outcome measurement, and result reporting. Judgments were “low,” “some concerns,” or “high” at the domain level, with an overall judgment based on criteria [8]. Moreover, it employs a macroenabled Excel file, released by the revised version of RoB2 on August 22, 2019, as an assessment tool [9]. If any discrepancies arise during the data extraction or assessment process, a third researcher would propose potential solutions.

### 2.4. Effect Measure

Outcome measures include the following: total adverse events, adverse drug-related reactions, alanine aminotransferase (ALT)/aspartate aminotransferase increase, blood creatine phosphokinase increase, myalgia, myopathy, muscle complaints, rhabdomyolysis, and gastrointestinal disorders. Total adverse events are defined as any harmful or unexpected symptoms or abnormal laboratory indicators, whether or not they are drug related. Adverse drug-related reactions are defined as adverse reactions associated with the drug. The numerical reference standards for ALT, AST, and CK are the upper limit of normal (ULN). They are classified based on values greater than 3 × ULN, 5 × ULN, or 10 × ULN. Symptoms self-reported by subjects during the trial, such as muscle pain, muscle weakness, and muscle stiffness, are classified using the “Regulatory Activities Medical Dictionary” (MedDRA) [10]. The ratio of the number of individuals in the experimental group experiencing various adverse reactions to the total number of individuals in the experimental group is used as the outcome measure.

### 2.5. Statistical Analysis

First, study data was grouped by main diagnoses; then, data for similar adverse reactions were combined.

Using R (4.3.1), a meta-analysis was conducted on adverse reaction proportions relative to the experimental group's participants. Before this, normality tests were performed on original and converted rates, selecting the most normalizing method. Combined estimates, including 95% confidence intervals, were given. The “incr” value (0.5) replaced zero event occurrences and was used across all studies. For heterogeneity, Cochran's *Q* test and *I*^2^ statistics were applied, with *p* > 0.05 indicating no heterogeneity, 25%–50% low, 50%–75% moderate, and > 75% high heterogeneity [11]. For heterogeneity assessment, a “leave-one-out” analysis was performed on the studies (Supporting Information 2: Figure S1, Supporting Information 3: Figure S2, Supporting Information 4: Figure S3, Supporting Information 5: Figure S4, Supporting Information 6: Figure S5, Supporting Information 7: Figure S6, Supporting Information 8: Figure S7, Supporting Information 9: Figure S8, Supporting Information 10: Figure S9, Supporting Information 11: Figure S10, Supporting Information 12: Figure S11, Supporting Information 13: Figure S12, Supporting Information 14: Figure S13, and Supporting Information 15: Figure S14). Then, a random effects model was applied to examine the effect of baseline covariates on heterogeneity. For *I*^2^ > 50%, subgroup analysis was conducted, including types and doses of statin drugs as covariates, with a forest plot generated (details in Supporting Information). A network meta-analysis was performed in Stata (Version 17.0) to compare results with significant heterogeneity. Using the frequency network analysis method, an optimal ranking probability graph was created, and the surface under the cumulative ranking curve (SUCRA) was used to evaluate the adverse reaction probabilities for various treatments.

## 3. Results

### 3.1. Eligible Studies and the Risks of Bias Assessment

**Figure 1:**
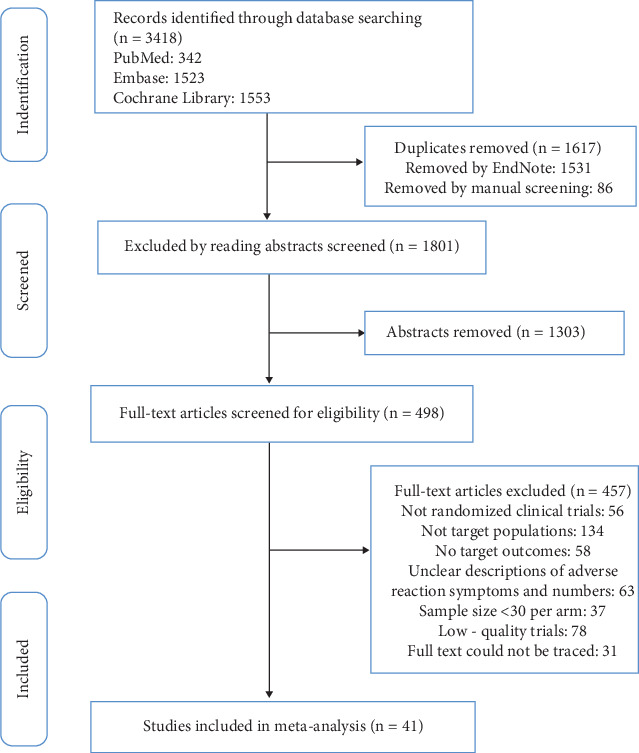
Flow diagram of study selection process.

Using a set search strategy, we scanned three databases and found 3418 studies. Duplicate entries were removed in EndNote software, and additional duplicates were eliminated by reviewing titles. Abstracts were then screened to exclude less relevant articles, and full texts were read to identify studies that met our inclusion and exclusion criteria. Ultimately, 41 studies involving 64,728 subjects were included [12–52]. The PRISMA flowchart depicting the acquisition process is presented in Figure 1. Among the 41 studies included, 19 targeted patients with hyperlipidemia, 8 focused on patients with coronary heart disease, 6 focused on patients with acute coronary syndrome or acute ischemic stroke, 2 focused on patients with chronic heart failure, and 6 focused on patients with diabetes. These studies encompassed six different types of statins at various dosages (atorvastatin, rosuvastatin, fluvastatin, pitavastatin, simvastatin, and pravastatin). Baseline characteristics of the included studies are displayed in Table 1.

**Figure 2:**
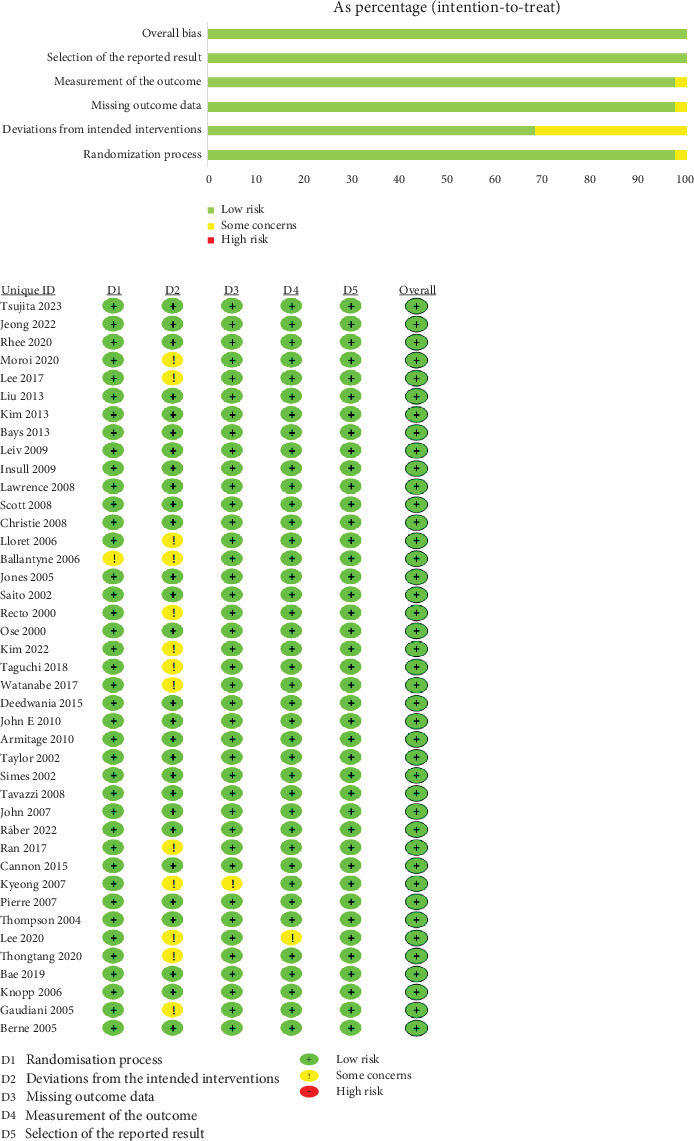
Risk of bias assessment.

All trials mentioned random allocation of participants as a means to mitigate bias risk. Specifically, 21 studies stated the methods used for generating random sequences [12, 13, 15, 17, 19–24, 30–32, 35–37, 39–42, 49], while three trials described allocation concealment [12, 22, 31]. The remaining studies did not provide detailed information on this aspect. Double-blind methods were employed in 28 studies [12–14, 17–20, 22–24, 27, 28, 30, 34–36, 38–41, 43–46, 49–52], and two studies mentioned outcome concealment [19, 21]. All studies correctly reported reasons for participant attrition and provided baseline data to assess comparability between groups.  Figure 2 provides a summary of the assessment of bias risk.

### 3.2. Meta-Analysis of the Prevalence of Adverse Reactions to Statins in Various Diseases

Due to the significant differences in adverse reactions among different disease populations and the varying emphasis on recording adverse reactions, the subjects were divided into five major groups: hyperlipidemia patients, coronary heart disease patients, acute coronary syndrome or acute ischemic stroke patients, heart failure patients, and diabetes mellitus patients for subgroup analysis. Additionally, according to the 2013 ACC/AHA guidelines, statins were categorized into high-, moderate-, and low-dose groups for subgroup analysis [53].

#### 3.2.1. The Adverse Reactions of Statins in Hyperlipidemia Patients

The six indicators with the highest reported frequency in safety evaluation for hyperlipidemia patients were as follows: total adverse events, adverse drug-related events, elevated ALT/aspartate aminotransferase level, elevated blood creatine phosphokinase level, myalgia, and gastrointestinal disorders.

##### 3.2.1.1. The Total Adverse Event Rate of Statins in Hyperlipidemia Patients

**Figure 3:**
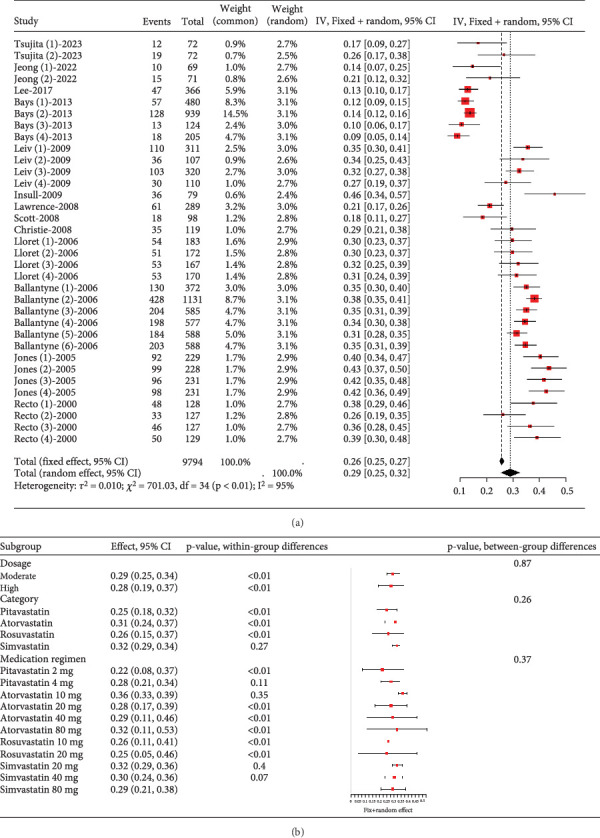
(a) The total adverse event rate of statins in hyperlipidemia patients. (b) Subgroup analysis of the total adverse event rate of statins in hyperlipidemia patients. The black diamond represents the pooled effect estimate (with 95% confidence interval) from the meta-analysis. The red box indicates individual study effect sizes (with box size proportional to study weight in the analysis). Horizontal lines show 95% confidence intervals for each study.

In hyperlipidemia patients taking statins, a total adverse event rate of about 0.29 (95% CI [0.25, 0.32], *p* < 0.01) was observed, with high heterogeneity (*I*^2^ = 95%) (Figure 3a). Postsubgroup analysis, no difference was found in total adverse event rates among pitavastatin (2 and 4 mg), atorvastatin (10, 20, 40, and 80 mg), rosuvastatin (10 and 20 mg), and simvastatin (20, 40, and 80 mg) (Figure 3b).

##### 3.2.1.2. Adverse Drug-Related Event Rate of Statins in Hyperlipidemia Patients

**Figure 4:**
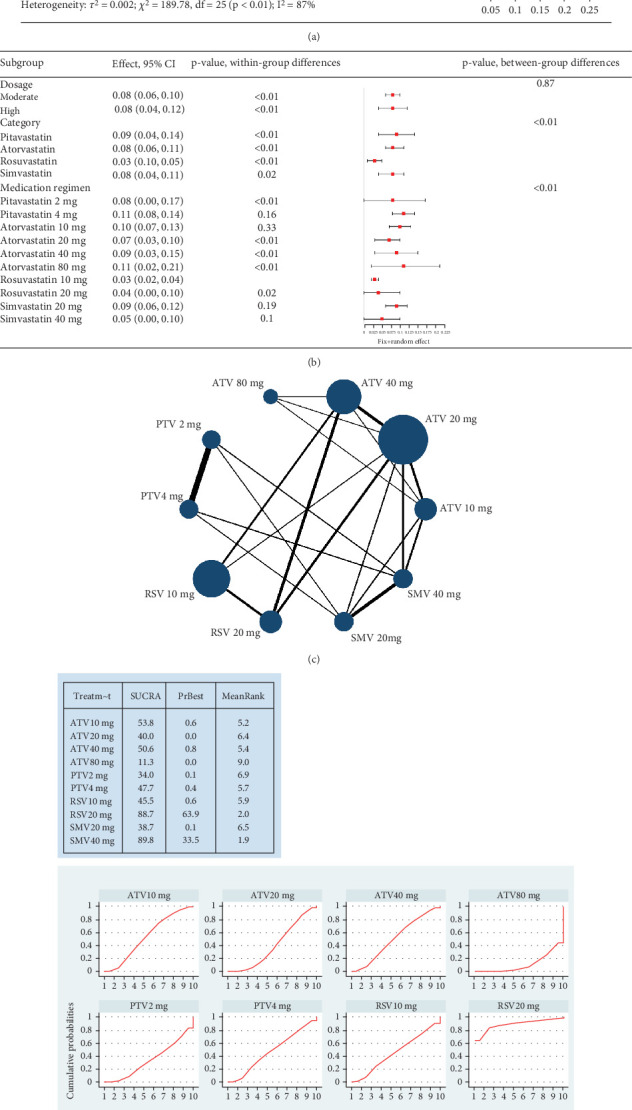
(a) The adverse drug-related event rate of statins in hyperlipidemia patients. (b) Subgroup analysis of the adverse drug-related event rate of statins in hyperlipidemia patients. The black diamond represents the pooled effect estimate (with 95% confidence interval) from the meta-analysis. The red box indicates individual study effect sizes (with box size proportional to study weight in the analysis). Horizontal lines show 95% confidence intervals for each study. (c) Network diagram. (d) Surface under the cumulative ranking curve plot. The surface under the cumulative ranking curve (SUCRA) value is the probability each treatment has of being among the best of those in the network, with larger values representing higher ranking probabilities. (e) League table for network meta-analysis. The table presents the results of pairwise comparisons of the incidence of the adverse drug-related event rate of statins (RD, risk difference), with 0 as the equivalent value for RD. When the 95% CI spans 0, it indicates that there is no statistically significant difference between the two. The symbol “▲” represents a statistically difference between the two drugs.

Hyperlipidemia patients had an adverse event rate of roughly 0.08 (95% CI [0.06, 0.09]), with high heterogeneity (*I*^2^ = 87%) (Figure 4a). Subgroup analysis indicated varied adverse event rates across statin types and dosages (*p* < 0.01) (Figure 4b). A network meta-analysis was conducted on studies that included two or more statin drugs (Figure 4c). The cumulative ranking curve shows that simvastatin 40 mg ranks best in terms of adverse drug-related events (Figure 4d). A consistency model was used for network meta-analysis, forming pairwise comparisons among 10 drug regimens, resulting in 45 comparisons. Among these, four comparisons showed statistical significance. These were the risk differences (RDs) in drug-related adverse reactions between simvastatin 40 mg and pitavastatin 4 mg, simvastatin 20 mg, pitavastatin 2 mg, and atorvastatin 80 mg, respectively.

##### 3.2.1.3. The Rate of Transaminase Elevation in Hyperlipidemia Patients Caused by Statins

**Figure 5:**
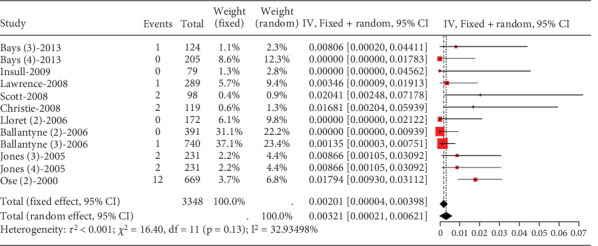
Transaminase elevation > 3× ULN of the high-dose group in hyperllipidemia patients. The black diamond represents the pooled effect estimate (with 95% confidence interval) from the meta-analysis. The red box indicates individual study effect sizes (with box size proportional to study weight in the analysis). Horizontal lines show 95% confidence intervals for each study.

In hyperlipidemia patients, transaminase elevation data did not adhere to normal distribution. Upon categorizing into high-dose and nonhigh-dose groups and statistical testing, only the high-dose group exhibited normal distribution. Meta-analysis on this group (rosuvastatin 20 mg, atorvastatin 40 and 80 mg, and simvastatin 80 mg) showed low transaminase elevation events (0.00201, 95% CI [0.00004, 0.00398]). Studies demonstrated low heterogeneity (*I*^2^ = 33%) (Figure 5).

##### 3.2.1.4. The Rate of Blood Creatine Phosphokinase Elevation in Hyperlipidemia Patients Caused by Statins

**Figure 6:**
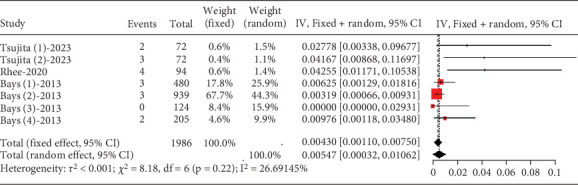
CK elevation > 3× ULN in hyperlipidemia patients. The black diamond represents the pooled effect estimate (with 95% confidence interval) from the meta-analysis. The red box indicates individual study effect sizes (with box size proportional to study weight in the analysis). Horizontal lines show 95% confidence intervals for each study.

About 0.43% (95% CI [0.0011, 0.0075]) of hyperlipidemia patients showed CK elevation, not exceeding three times the upper limit, with low study heterogeneity (*I*^2^ = 27%) (Figure 6). For CK elevation exceeding 5 and 10 times the upper limit, a normal distribution could not be assumed, hindering meta-analysis.

##### 3.2.1.5. The Rate of Myalgia in Hyperlipidemia Patients Caused by Statins

**Figure 7:**
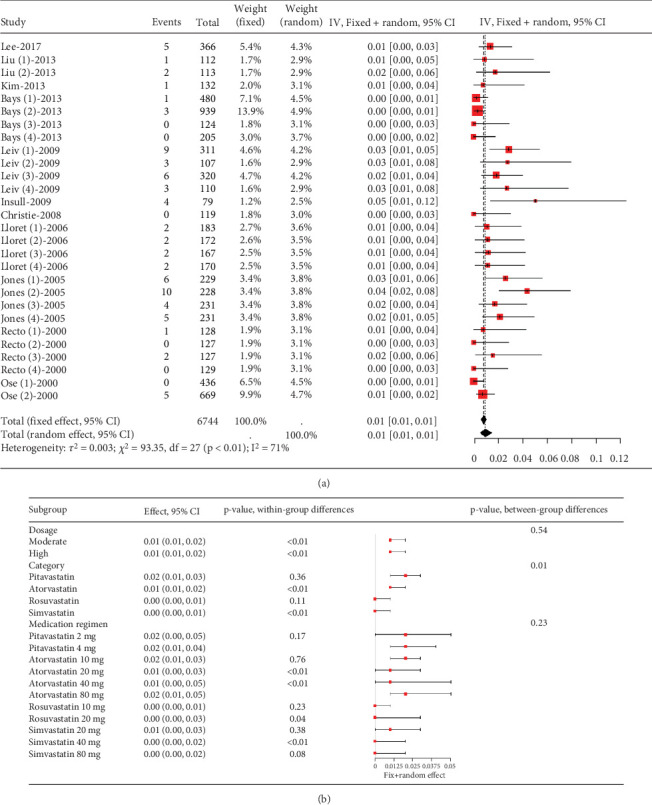
(a) The rate of myalgia in hyperlipidemia patients caused by statins. (b) Subgroup analysis of the rate of myalgia in hyperlipidemia patients. The black diamond represents the pooled effect estimate (with 95% confidence interval) from the meta-analysis. The red box indicates individual study effect sizes (with box size proportional to study weight in the analysis). Horizontal lines show 95% confidence intervals for each study.

The incidence of statin-induced myalgia is about 0.01 (95% CI [0.01, 0.01]), showing moderate heterogeneity (*I*^2^ = 61%) (Figure 7a). Analysis found no difference in myalgia rates between moderate and high statin doses (*p* = 0.54) nor among various statin types and dosages (*p* = 0.23) (Figure 7b).

##### 3.2.1.6. The Rate of Gastrointestinal Disorders in Hyperlipidemia Patients Caused by Statins

**Figure 8:**
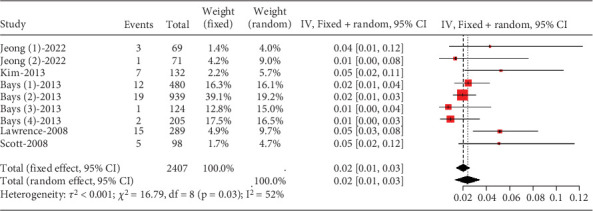
The rate of gastrointestinal disorder in hyperlipidemia patients. The black diamond represents the pooled effect estimate (with 95% confidence interval) from the meta-analysis. The red box indicates individual study effect sizes (with box size proportional to study weight in the analysis). Horizontal lines show 95% confidence intervals for each study.

The proportion of hyperlipidemia patients experiencing gastrointestinal disorders was approximately 0.02 (95% CI [0.00, 0.03]), with moderate heterogeneity (*I*^2^ = 52%) (Figure 8).

#### 3.2.2. The Adverse Reactions of Statins in Coronary Heart Disease Patients

The four indicators with the highest reported frequency in safety evaluation for coronary heart disease patients were as follows: elevated ALT/aspartate aminotransferase, myalgia, rhabdomyolysis, and myopathy.

##### 3.2.2.1. The Rate of Transaminase Elevation in Coronary Heart Disease Patients Caused by Statins

**Figure 9:**
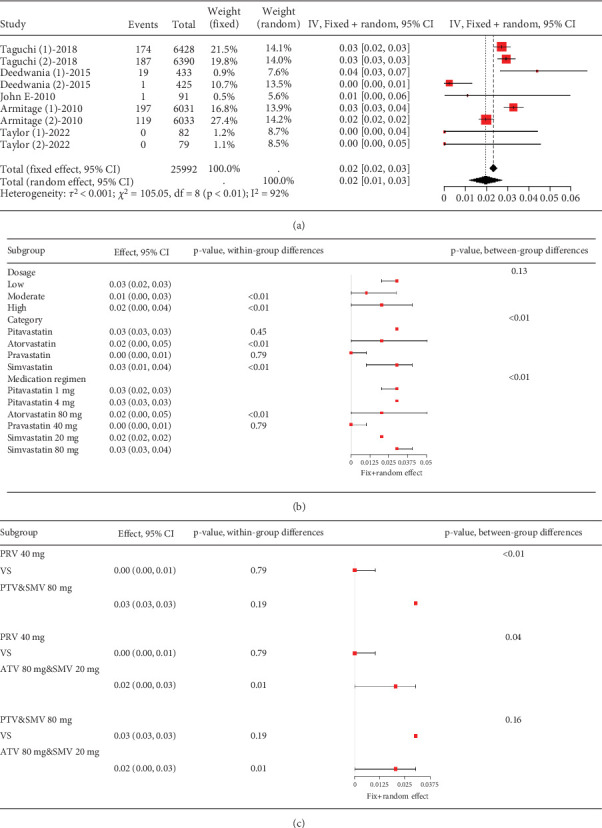
(a) Transaminase elevation > 3× ULN in coronary heart disease patients. (b) Subgroup analysis of transaminase elevation in coronary heart disease patients. (c) Pairwise comparisons of transaminase elevation rates among three group statins. The black diamond represents the pooled effect estimate (with 95% confidence interval) from the meta-analysis. The red box indicates individual study effect sizes (with box size proportional to study weight in the analysis). Horizontal lines show 95% confidence intervals for each study.

Remarkable variation was observed in the proportion of coronary heart disease patients with transaminase levels over three times the upper limit value (*I*^2^ = 92%, Figure 9a). Subgroup analysis revealed that different statin types and dosages led to varying outcomes (*p* < 0.01), with pravastatin showing lower transaminase elevation rates (Figure 9b). Notably, the pravastatin 40 mg group had a remarkably lower transaminase elevation rate compared to pitavastatin and simvastatin 80 mg, as well as atorvastatin 80 mg and simvastatin 20 mg (*p* < 0.01 and *p* = 0.04, respectively). However, difference was not found between the pitavastatin and simvastatin 80 mg and atorvastatin 80 mg and simvastatin 20 mg groups (*p* = 0.16) (Figure 9c).

##### 3.2.2.2. The Rate of Myalgia in Coronary Heart Disease Patients Caused by Statins

**Figure 10:**
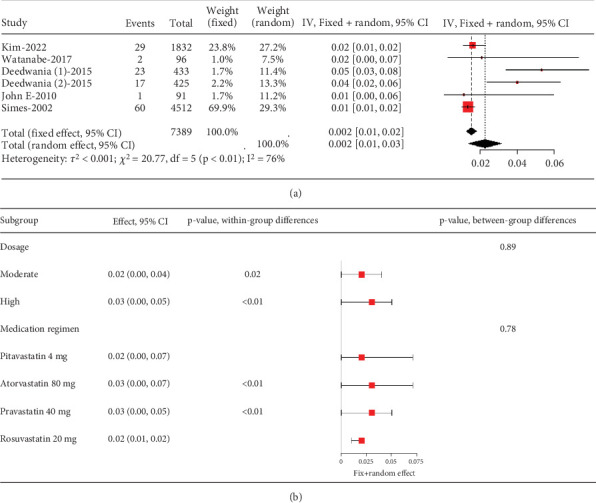
(a) The rate of myalgia in coronary heart disease patients caused by statins. (b) Subgroup analysis of the rate of myalgia in coronary heart disease patients. The black diamond represents the pooled effect estimate (with 95% confidence interval) from the meta-analysis. The red box indicates individual study effect sizes (with box size proportional to study weight in the analysis). Horizontal lines show 95% confidence intervals for each study.

The rate of myalgia in coronary heart disease patients shows a remarkable variability across studies (*I*^2^ = 76%, Figure 10a), but subgroup analysis did not find differences in myalgia probability between moderate- and high-dose groups (*p* = 0.89) or among various statin types and dosages (*p* = 0.78) (Figure 10b).

##### 3.2.2.3. The Rate of Myopathy in Coronary Heart Disease Patients Caused by Statins

**Figure 11:**
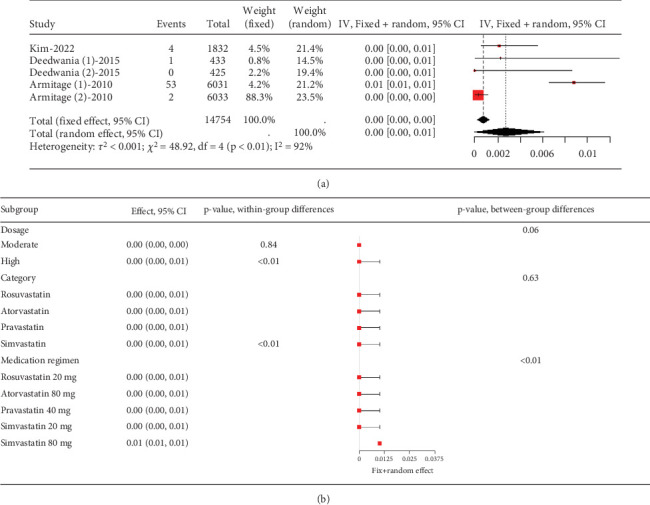
(a) The rate of myopathy in coronary heart disease patients caused by statins. (b) Subgroup analysis of the rate of myopathy in coronary heart disease patients caused by statins. The black diamond represents the pooled effect estimate (with 95% confidence interval) from the meta-analysis. The red box indicates individual study effect sizes (with box size proportional to study weight in the analysis). Horizontal lines show 95% confidence intervals for each study.

The rate of myopathy in coronary heart disease patients varies remarkably (*I*^2^ = 92%, Figure 11a), with subgroup analysis indicating that simvastatin 80 mg notably increases myopathy risk compared to other groups (*p* < 0.01) (Figure 11b).

##### 3.2.2.4. The Rate of Rhabdomyolysis in Coronary Heart Disease Patients Caused by Statins

**Figure 12:**
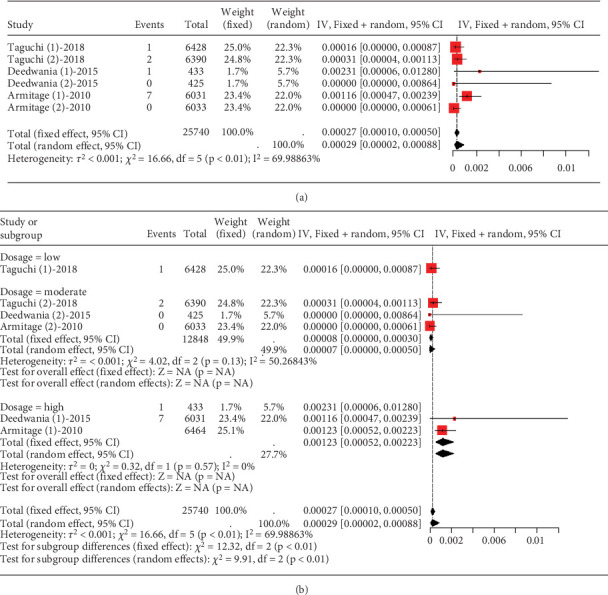
(a) The rate of rhabdomyolysis in coronary heart disease patients caused by statins. (b) Subgroup analysis of the rate of rhabdomyolysis in coronary heart disease patients. The black diamond represents the pooled effect estimate (with 95% confidence interval) from the meta-analysis. The red box indicates individual study effect sizes (with box size proportional to study weight in the analysis). Horizontal lines show 95% confidence intervals for each study.

The data on rhabdomyolysis in coronary heart disease patients showed a remarkable heterogeneity (*I*^2^ = 70%, Figure 12a). Subgroup analysis revealed that the rhabdomyolysis incidence correlated with dosage, with clear differences noted between low-, moderate-, and high-dose groups (*p* < 0.01); the rates of rhabdomyolysis for the low-, moderate-, and high-dose groups were 0.00016 (95% CI [0.0000, 0.00087]), 0.00007 (95% CI [0.0000, 0.0005]), and 0.00123 (95% CI [0.00052, 0.00223]), respectively, and no difference within the moderate- and high-dose subgroups (*p* = 0.13 and *p* = 0.57) (Figure 12b).

#### 3.2.3. The Adverse Reactions of Statins in Heart Failure Patients

**Figure 13:**
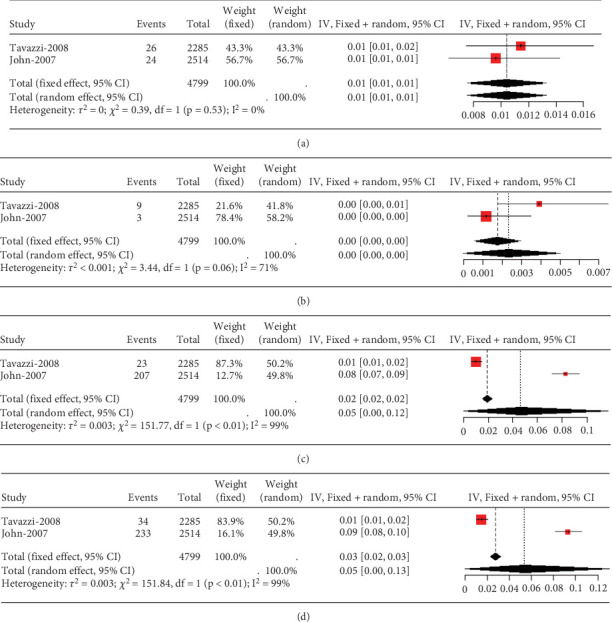
(a) Transaminase elevation > 3× ULN in heart failure patients. (b) CK elevation > 10× ULN in heart failure patients. (c) The rate of myalgia in heart failure patients caused by statins. (d) The rate of gastrointestinal disorders in heart failure patients caused by statins. The black diamond represents the pooled effect estimate (with 95% confidence interval) from the meta-analysis. The red box indicates individual study effect sizes (with box size proportional to study weight in the analysis). Horizontal lines show 95% confidence intervals for each study.

Two studies on heart failure patients treated with 10 mg rosuvastatin noted safety issues like aminotransferase/aspartate aminotransferase, elevated blood creatine phosphokinase, myalgia, and gastrointestinal disorders. Only transaminase elevation over three times the normal value showed no study-to-study variation (*I*^2^ = 0%, Figure 13a). However, heterogeneity was remarkable for CK exceeding 10 times the ULN value, myalgia, and gastrointestinal disorders (*I*^2^ = 71%, 99%, and 99%, respectively, Figures 13b, 13c, and 13d).

#### 3.2.4. The Adverse Reactions of Statins in Acute Coronary Syndrome or Acute Stroke Patients

In studies involving patients with acute coronary syndrome or acute stroke, the safety evaluation mentioned two frequently observed indicators: elevated transaminases and myalgia.

##### 3.2.4.1. The Rate of Transaminase Elevation in Acute Coronary Syndrome or Acute Ischemic Stroke Patients Caused by Statins

**Figure 14:**
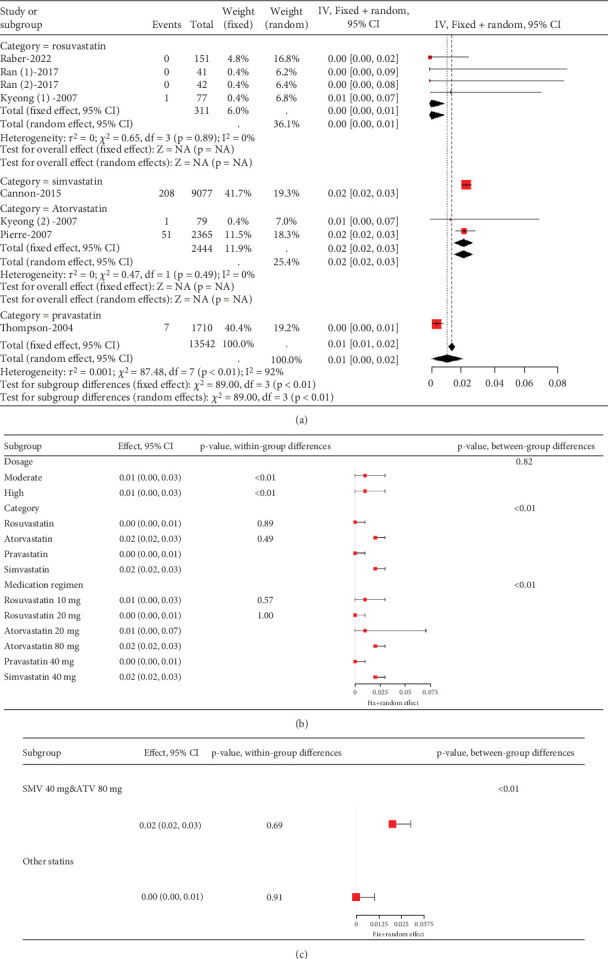
(a) Transaminase elevation > 3× ULN in ACS or CIS patients. (b) Subgroup analysis of transaminase elevation > 3× ULN in ACS or CIS patients. (c) Comparison of the rate of transaminase elevation > 3× ULN among the three statin groups. The black diamond represents the pooled effect estimate (with 95% confidence interval) from the meta-analysis. The red box indicates individual study effect sizes (with box size proportional to study weight in the analysis). Horizontal lines show 95% confidence intervals for each study.

Remarkable heterogeneity (*I*^2^ = 92%) was observed in the transaminase elevation exceeding three times the ULN (Figure 14a). Subgroup analysis revealed similar transaminase elevation rates between 40 mg simvastatin and 80 mg atorvastatin (Figure 14b). Consequently, data from these two groups were combined for effect size estimation, compared with others, showing higher transaminase elevation rates for simvastatin 40 mg and atorvastatin 80 mg compared to 10 and 20 mg rosuvastatin, 20 mg atorvastatin, and 40 mg pravastatin (*p* < 0.01). There was no statistically difference within these two groups (*p* = 0.69 and *p* = 0.91) (Figure 14c).

##### 3.2.4.2. The Rate of Myalgia in Acute Coronary Syndrome or Acute Ischemic Stroke Patients Caused by Statins

**Figure 15:**
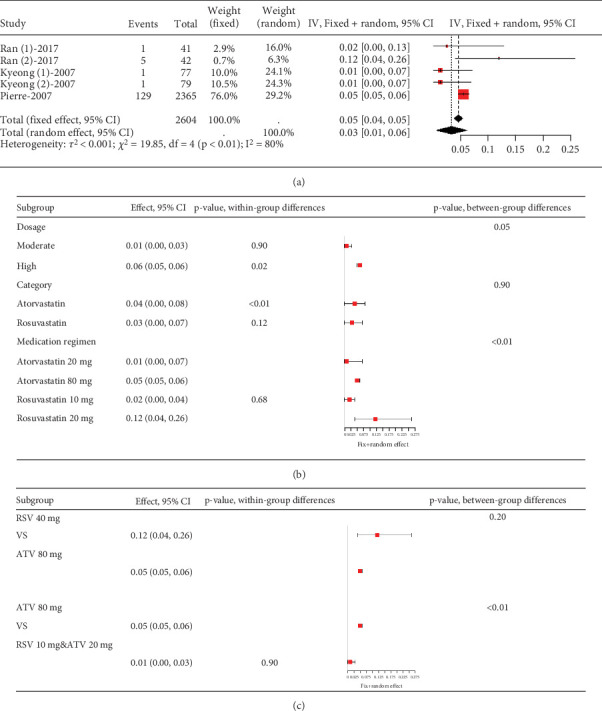
(a) The rate of myalgia in ACS or CIS patients caused by statins. (b) Subgroup analysis of the rate of myalgia in ACS or CIS patients. (c) Comparison of the rate of myalgia in ACS or CIS patients between three group statins. The black diamond represents the pooled effect estimate (with 95% confidence interval) from the meta-analysis. The red box indicates individual study effect sizes (with box size proportional to study weight in the analysis). Horizontal lines show 95% confidence intervals for each study.

Statins' myalgia occurrence varies greatly across studies (*I*^2^ = 96%, Figure 15a). Subgroup analysis indicates that the 20 mg rosuvastatin and 80 mg atorvastatin doses increase myalgia risk compared to 10 mg rosuvastatin and 20 mg atorvastatin (Figure 15b). Combining 10 mg rosuvastatin and 20 mg atorvastatin data for analysis showed no difference in the myalgia rate between 20 mg rosuvastatin and 80 mg atorvastatin (*p* = 0.20). Notably, the 80 mg atorvastatin dose resulted in a higher myalgia rate than both 10 mg rosuvastatin and 20 mg atorvastatin (*p* < 0.01, Figure 15c).

#### 3.2.5. The Adverse Reactions of Statins in Diabetes Mellitus Patients

In studies involving patients with diabetes mellitus, the safety evaluation mentioned two frequently observed indicators: elevated transaminases and myalgia.

##### 3.2.5.1. The Rate of Transaminase Elevation in Diabetes Mellitus Patients Caused by Statins

**Figure 16:**
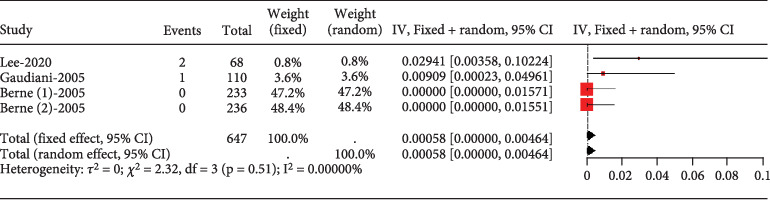
Transaminase elevation > 3× ULN in diabetes mellitus patients. The black diamond represents the pooled effect estimate (with 95% confidence interval) from the meta-analysis. The red box indicates individual study effect sizes (with box size proportional to study weight in the analysis). Horizontal lines show 95% confidence intervals for each study.

The proportion of diabetic patients experiencing transaminase elevation over three times the upper normal limit was rare (0.00058, 95% CI [0.00000, 0.00464]), showing no study heterogeneity (*I*^2^ = 0%) (Figure 16).

##### 3.2.5.2. The Rate of Myalgia in Diabetes Mellitus Patients Caused by Statins

**Figure 17:**
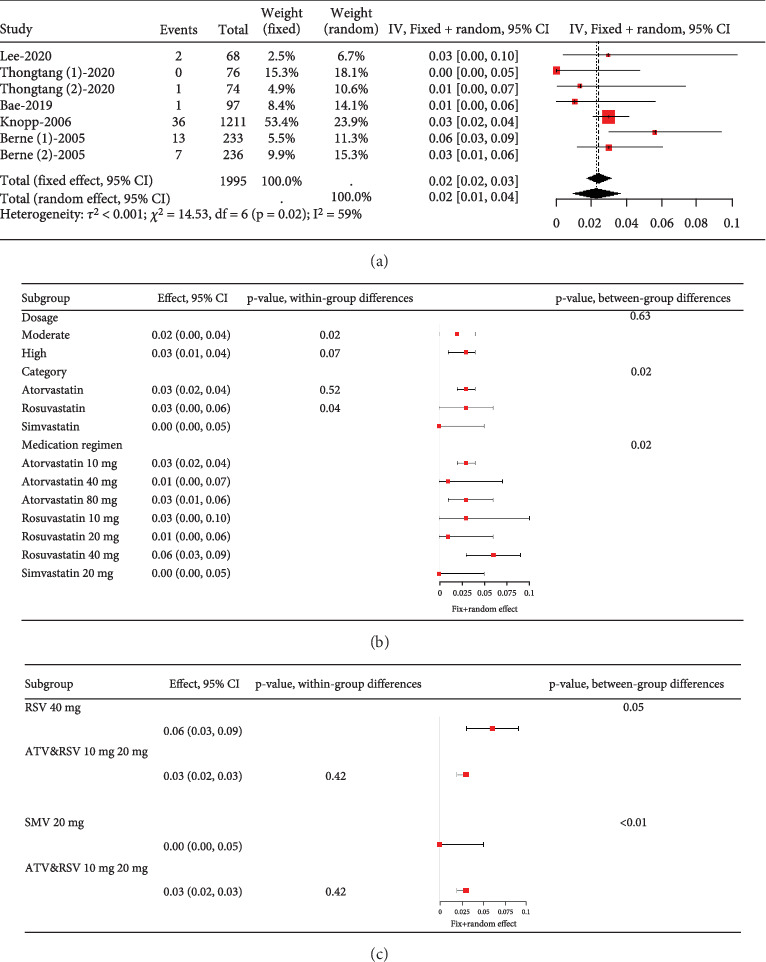
(a) The rate of myalgia in diabetes mellitus patients caused by statins. (b) Subgroup analysis of the rate of myalgia in diabetes mellitus patients. (c) Comparison of the rate of myalgia in diabetes mellitus patients between three group statins. The black diamond represents the pooled effect estimate (with 95% confidence interval) from the meta-analysis. The red box indicates individual study effect sizes (with box size proportional to study weight in the analysis). Horizontal lines show 95% confidence intervals for each study.

Analysis of studies in diabetic patients showed high heterogeneity for myalgia occurrence (*I*^2^ = 96%, Figure 17a). Subgroup analysis indicated that 40 mg rosuvastatin had a higher myalgia rate than others, while 20 mg simvastatin had a lower rate (Figure 17b). In order to clarify whether the difference was caused by rosuvastatin 40 mg or simvastatin 20 mg, comparisons were made between rosuvastatin 40 mg and simvastatin 20 mg with the other groups, respectively. Comparisons revealed no difference in the myalgia rate between 40 mg rosuvastatin and atorvastatin 10, 40, and 80 mg, or rosuvastatin 20 mg (*p* = 0.05). In contrast, 20 mg simvastatin had different myalgia rates compared to the aforementioned groups (*p* < 0.01), with no variation in the atorvastatin 10, 40, and 80 mg and rosuvastatin 20 mg groups (*p* = 0.42) (Figure 17c).

## 4. Discussion

Past studies mainly examined specific adverse effects of statin treatment on individual diseases. A gap exists for a broader analysis encompassing various adverse effects from using different statins at various doses across multiple diseases. To investigate if the risk of experiencing adverse effects varies among patients with diverse diseases on statins, we performed a thorough review of clinical trials on statin-induced side effects, enrolling 64,728 participants.

In our hyperlipidemia study, we found that moderate and high doses of statins resulted in similar rates of total adverse effects, such as myalgia, elevated transaminases, blood creatine phosphokinase, and gastrointestinal issues. Network meta-analysis showed that simvastatin 40 mg had the lowest rate of drug-related adverse reactions compared to pitavastatin 4 and 2 mg, as well as atorvastatin 80 mg. This suggests that high-dose statins may lead to fewer adverse reactions in patients with limited comorbidities. This conclusion was reinforced by a study on healthy individuals, which demonstrated that 6 months of 80 mg atorvastatin did not reduce average muscle strength or exercise performance [54]. Moreover, it has been found that the use of high doses of statins in patients with hyperlipidemia does not lead to a significant increase in transaminases, indicating the safety of statins for the liver. This conclusion was supported by a clinical study conducted in 2012 aimed at assessing the safety and efficacy of atorvastatin and pitavastatin in patients with mild to moderate elevations in liver enzymes. In this study, 135 patients with hypercholesterolemia, whose serum ALT levels were elevated to 1.25–2.5 times the ULN (without alcoholic or viral hepatitis), continued to use statins. After a 12-week observation period, their transaminase levels did not continue to rise and even showed a decrease [55]. This is because statins can alter cell regulation processes by activating peroxisome proliferator–activated receptors, inhibiting fatty acid beta-oxidation, reducing inflammatory mediators, and blocking the sonic hedgehog pathway, thereby inducing the resolution of inflammation and fat deposition in the liver [56].

The incidence of transaminase elevation varies, but 40 mg pravastatin shows the lowest rate compared to other statins in patients with coronary heart disease. For myopathy, 80 mg simvastatin has a notably higher rate than other doses. Myalgia and rhabdomyolysis occurrence is similar between moderate and high doses of statins. However, 80 mg simvastatin has a higher incidence of muscle damage, leading to its US FDA restriction in June 2011 [57]. A genome-wide association study published in the *New England Journal of Medicine* in 2008 which used 40 and 80 mg of simvastatin found a strong link between myopathy and the rs4363657 SNP in the SLCO1B1 gene on Chromosome 12 [58]. Nevertheless, a genome-wide association study conducted in 2013 with rosuvastatin found no increased risk of myalgia in its users carrying the rs4363657C or rs4149056C alleles in the SLCO1B1 gene [59]. Whether there are differences in the interaction between different statins and allele genes on chromosomes requires further investigation.

Two studies on heart failure patients treated with 10 mg rosuvastatin showed no difference in elevated transaminases, but the elderly group had higher rates of CK levels exceeding 10 times the ULN, muscle symptoms, and gastrointestinal issues. This suggests that advanced age and comorbidities increase adverse reactions to statins. Pharmacokinetic studies confirmed that rosuvastatin boosts endothelial nitric oxide (NO) production, alleviates myocardial damage, and improves variability (HRV) and blood pressure variability (BPV) by reducing caveolin-1 expression and enhancing apolipoprotein (apo) E-/- and NO synthase (NOS) function [60, 61]. The JUPITER trial prospectively assessed rosuvastatin 20 mg's efficacy, revealing its capability to reduce cardiovascular events in healthy individuals [62]. Rosuvastatin faced scrutiny, with a public petition on March 4, 2004, seeking its removal from the US market [63]. In the same year, *The Lancet* pointed out that 80 mg rosuvastatin showed severe myopathy and rhabdomyolysis in clinical trials [64]. Approval for rosuvastatin's 80 mg dose was put on hold pending the conclusion of the multinational safety assessment program [65]. Following the controversy, 80 mg rosuvastatin was scarcely used in clinical trials. Limited reports exist on its adverse effects. Considering rosuvastatin's pharmacokinetic profile and our study results, it is safe for elderly cardiac patients, with minimal liver impact, at moderate doses.

Statin type and dosage notably affect two adverse reactions in patients with acute coronary syndrome or acute ischemic stroke: elevated transaminases and myalgia. Higher doses of simvastatin and atorvastatin show a higher risk of transaminase elevation compared to lower doses of rosuvastatin and pravastatin. Also, high-dose statins have a higher likelihood of causing myalgia. In long-term studies, the rate of persistent transaminase elevation with atorvastatin 80 mg or simvastatin exceeds that in other groups [66]. A large-scale data meta-analysis study published in *The Lancet* in 2022 found high-intensity statin treatments (40–80 mg atorvastatin and 20–40 mg rosuvastatin daily) had a higher relative risk for muscle symptoms compared to low or moderate intensity regimens [67]. Therefore, for patients with acute coronary syndrome or acute ischemic stroke needing high-intensity statins, 80 mg atorvastatin or simvastatin should be used cautiously in those with impaired hepatic function.

In diabetes patients, statin use showed no variation in transaminase elevation. High-dose statins (40 mg atorvastatin, 80 mg atorvastatin, 20 mg rosuvastatin, and 40 mg rosuvastatin) and 20 mg simvastatin had similar myalgia rates. High-dose atorvastatin and rosuvastatin offer efficacy with minimized muscle side effects, aligning with other studies showing comparable myopathy and rhabdomyolysis incidence with 80 mg atorvastatin to lower doses [65]. However, in September 2013, the FDA advised reducing the maximum daily atorvastatin dose to 40 mg, down from 80 mg, following a reassessment of clinical data that highlighted an elevated risk of myopathy with higher doses.

The effects of statins on the liver are generally similar; however, in patients with coronary heart disease, it has been observed that pravastatin has a lower rate of causing transaminase elevations compared to other statins. This may be attributed to pravastatin's effective transport and elimination through transport proteins associated with hepatic uptake and biliary excretion, along with its specific distribution in the liver. Furthermore, statins that are metabolized by the cytochrome P450 enzyme system are susceptible to metabolism-mediated drug–drug interactions. Pravastatin undergoes very little metabolism by P450 and, therefore, is not prone to drug–drug interactions involving metabolism [68]. This also explains the lack of significant impact on transaminase levels in elderly patients treated with rosuvastatin, which shares a similar mechanism of action to pravastatin under equivalent dosing conditions.

In contrast, simvastatin and atorvastatin, which rely on the cytochrome P450 system for metabolism, show relatively fewer adverse drug reactions in hyperlipidemic patients with fewer comorbidities. However, in patients with multiple comorbidities (and therefore more medications) such as those with acute coronary syndrome or acute ischemic stroke, caution should be exercised when using simvastatin or high-dose atorvastatin (80 mg) due to impaired liver metabolism. This precaution may arise from the fact that simvastatin, being a lipophilic statin in its lactone form, easily crosses cellular membranes for intestinal absorption. It is also a high-affinity substrate for CYP3A4 and P-glycoprotein (P-gp), which, in combination with other drugs, may lead to detoxification in the intestine, resulting in lower bioavailability. Additionally, the inhibition of CYP3A4 and/or P-gp may increase absorption and lead to drug interactions [68].

## 5. Conclusion

Statins' side effects differ across patient groups. For those with hypercholesterolemia and diabetes, various statins have a similar impact on transaminase levels. However, patients with coronary heart disease, acute coronary syndrome, or stroke see differing effects. Notably, myalgia risk in hypercholesterolemia patients taking different statins aligns. Yet, in those with acute coronary syndrome or stroke, high-dose statin users face a higher myalgia risk compared to those on moderate or low doses.

## Figures and Tables

**Table 1:** Baseline characteristics of the included studies.

**Study (author, year)**	**Country/region, population**	**Intervention (per day)**	**Sample size**	**Mean age (mean ± SD, year)**	**Male sex (%)**	**Duration**	**Outcomes**
Tsujita 2023	Japan Patients with hypercholesterolemia	Pitavastatin 2 mg Pitavastatin 4 mg	72 72	56.4 ± 9.5 55.7 ± 8.5	34 (47.2%) 34 (47.2%)	12 weeks	Adverse events Adverse drug reactions ALT/AST increased CK increased > 10× ULN

Jeong 2022	Korea Patients with hypercholesterolemia	Pitavastatin 2 mg Pitavastatin 4 mg	69 70	64.1 ± 7.9 62.9 ± 9.9	44 (63.8%) 43 (61.4%)	8 weeks	Adverse events Adverse drug reactions ALT/AST increased > 3× ULN Musculoskeletal and connective tissue disorders Arthralgia Myopathy Gastrointestinal disorders

Rhee 2020	Korea Patients with hypertension and hypercholesterolemia	Rosuvastatin 20 mg	91	61.74 ± 9.75	65 (71.43%)	8 weeks	Adverse drug reactions CK increased

Moroi 2020	Japan Patients with hypercholesterolemia	Pitavastatin 2 mg Atorvastatin 10 mg	312 310	65.3 ± 10.1 65.4 ± 9.4	168 (53.8%) 168 (54.2%)	240 weeks	Rhabdomyolysis Muscle complaints New onset of diabetes mellitus ALT/AST increased > 3× ULN CK increased > 5× ULN

Lee 2017	Korea Patients with hypercholesterolemia	Atorvastatin 20 mg	366	63.3 ± 8.4	152 (45.1%)	8 weeks	Adverse events Adverse drug reactions Myalgia Lower extremity weakness Arthralgia

Liu 2013	China Patients with hypercholesterolemia and high risk of CHD	Pitavastatin 2 mg Atorvastatin 10 mg	112 113	58.7 ± 9.3 58.7 ± 7.9	69 (61.6%) 70 (61.9%)	12 weeks	Myalgia Back pain

Kim 2013	Korea Patients with hypercholesterolemia	Simvastatin 20 mg	132	58.6 ± 8.9	55 (44.7%)	8 weeks	Adverse drug reactions Musculoskeletal/connective tissue disorders Arthralgia Myalgia Gastrointestinal disorders Dyspepsia

Bays 2013	The United States, Canada, and Europe Patients with hypercholesterolemia and high risk of CHD	Atorvastatin 20 mg Rosuvastatin 10 mg Atorvastatin 40 mg Rosuvastatin 20 mg	483 944 126 206	59.6 ± 10.2 59.9 ± 9.7 58.2 ± 10.9 57.6 ± 10.1	230 (47.6%) 455 (48.2%) 63 (50.0%) 107 (51.9%)	12 weeks	Adverse events Adverse drug reactions ALT/AST increased > 3× ULN Gastrointestinal disorder myalgia

Leiv 2009	Russia, Norway, the United Kingdom, Finland, and Italy Patients with hypercholesterolemia or dyslipidemia	Pitavastatin 2 mg Simvastatin 20 mg Pitavastatin 4 mg Simvastatin 40 mg	315 108 323 111	58.7 ± 8.9 58.6 ± 9.6 57.7 ± 9.0 58.4 ± 9.5	115 (37.0%) 44 (41.1%) 125 (39.1%) 48 (43.6%)	12 weeks	Adverse events Adverse drug reactions ALT/AST increased CK increased > 5× ULN Myalgia

Insull 2009	The United States Patients with dyslipidemia	Atorvastatin 40 mg	79	51.5 ± 11.1	41 (51.9%)	12 weeks	Adverse events Adverse drug reactions ALT/AST increased > 3× ULN CK increased > 10× ULN Myalgia

Lawrence 2008	The United States and Canada Patients with hypercholesterolemia and high risk of CHD	Atorvastatin 80 mg	289	62 ± 9	178 (61%)	6 weeks	Adverse events Adverse drug reactions ALT/AST increased > 3× ULN CK increased > 10× ULN Gastrointestinal disorders

Scott 2008	The United States, Canada, Austria, etc. Patients with hypercholesterolemia and high risk of CHD	Atorvastatin 40 mg	98	58 ± 10	49 (50%)	6 weeks	Adverse events Adverse drug reactions ALT/AST increased > 3× ULN CK increased > 10× ULN Gastrointestinal disorders

Christie 2008	The United States High-risk patients with dyslipidemia	Simvastatin 80 mg	119	60	63 (51.2%)	24 weeks	Adverse events ALT/AST increased > 3× ULN CK increased > 10× ULN Myalgia Back pain Muscle cramp

Lloret 2006	The United States Hispanic Americans with hypercholesterolemia	Rosuvastatin 10 mg Rosuvastatin 20 mg Atorvastatin 10 mg Atorvastatin 20 mg	183 172 167 170	58.0 ± 10.8 57.8 ± 10.9 56.7 ± 11.7 59.0 ± 9.8	89 (48%) 84 (49%) 84 (50%) 88 (51%)	6 weeks	Adverse events ALT/AST increased > 3× ULN CK increased > 10× ULN Myalgia

Ballantyne 2006	The United States, Canada, Argentina, etc. High-risk patients with dyslipidemia	Rosuvastatin 10 mg Rosuvastatin 20 mg Atorvastatin 10 mg Atorvastatin 20 mg Simvastatin 20 mg Simvastatin 40 mg	372 1131 585 577 588 588	NA	NA	16 weeks	Adverse events ALT/AST increased > 3× ULN CK increased > 10× ULN Rhabdomyolysis

Jones 2005	The United States Patients with dyslipidemia	Atorvastatin 10 mg Atorvastatin 20 mg Atorvastatin 40 mg Atorvastatin 80 mg	229 228 231 231	61.5 ± 11.1 62.1 ± 11.1 61.6 ± 10.8 59.9 ± 11.5	139 (60.7%) 133 (58.3%) 141 (61.0%) 131 (56.7%)	8 weeks	Adverse events Adverse drug reactions ALT/AST increased > 3× ULN CK increased > 10× ULN Myalgia

Saito 2002	Japan Patients with hypercholesterolemia	Pitavastatin 2 mg Pravastatin 10 mg	124 109	57.5 58.8	40 (32%) 40 (36%)	12 weeks	ALT/AST increased > 3× ULN CK increased > 10× ULN

Recto 2000	Fourteen countries Patients with hypercholesterolemia	Simvastatin 20 mg Simvastatin 40 mg Atorvastatin 10 mg Atorvastatin 20 mg	128 127 127 129	Unclear	Unclear	12 weeks	Adverse events Adverse drug reactions ALT/AST increased > 3× ULN CK increased > 10× ULN Myalgia

Ose 2000	America, Europe, and Asia Patients with hypercholesterolemia	Simvastatin 40 mg Simvastatin 80 mg	436 669	52.7 ± 11.5 52.9 ± 10.8	243 (55.7%) 397 (59.3%)	48 weeks	ALT/AST increased > 3× ULN Myalgia Myopathy

Kim 2022	Korea Patients with atherosclerotic cardiovascular disease	Rosuvastatin 20 mg	1832	64 ± 10	1406 (75%)	3 years	Adverse drug reactions Gastrointestinal symptoms ALT/AST increased CK increased Fasting glucose concentration elevation New-onset diabetes Myalgia Myopathy Myonecrosis Gallbladder-related adverse events

Taguchi 2018	Japan Patients with coronary artery disease	Pitavastatin 1 mg Pitavastatin 4 mg	6428 6390	68.1 ± 8.3 68.0 ± 8.3	5124 (82.5%) 5129 (82.7%)	4 years	Rhabdomyolysis Muscle complaints Gallbladder-related events New onset of diabetes mellitus ALT/AST increased > 3× ULN CK increased > 5× ULN

Watanabe 2017	Japan Patients with coronary heart disease	Pitavastatin 4 mg	117	68 ± 10	81 (84%)	8 months	ALT/AST increased CK increased Myalgia

Deedwania 2015	South Africa Patients with coronary artery disease	Atorvastatin 80 mg Pravastatin 40 mg	433 425	72.4 ± 5 72.6 ± 5.2	299 (69.0%) 296 (69.6%)	1 year	ALT/AST increased > 3× ULN Myalgia Myopathy Rhabdomyolysis

John E 2010	Thirteen countries Patients with stable angina and coronary artery disease	Atorvastatin 80 mg	91	62.5 ± 8.6	NA	24 weeks	ALT/AST increased > 3× ULN CK increased Myalgia

Armitage 2010	United Kingdom Patients with previous myocardial infarction	Simvastatin 80 mg Simvastatin 20 mg	6031 6033	64.2 ± 8.9 64.2 ± 8.9	NA	6.7 ± 1.5 years	ALT/AST increased > 3× ULN CK increased > 5× ULN CK increased > 10× ULN Rhabdomyolysis Myalgia

Taylor 2002	The United States Patients with atherosclerotic cardiovascular disease	Pravastatin 40 mg Atorvastatin 80 mg	82 79	61 ± 12 58 ± 11	61 (74.4%) 54 (68.4%)	12 months	ALT/AST increased > 3× ULN Myositis

Simes 2002	Australia and New Zealand Patients with atherosclerotic cardiovascular disease	Pravastatin 40 mg	4512	62	3253 (83%)	6 years	Myositis or myalgia Gastrointestinal disorders

Tavazzi 2008	Italy Patients with chronic heart failure	Rosuvastatin 10 mg	2285	68 ± 11	1742 (76.2%)	12 months	Adverse drug reactions ALT/AST increased > 3× ULN CK increased > 5× ULN CK increased > 10× ULN Gastrointestinal disorders Muscle-related symptoms Rhabdomyolysis

John 2007	The United States Patients with chronic heart failure	Rosuvastatin 10 mg	2514	73 ± 7.1	1921 (76%)	35 months	ALT/AST increased > 3× ULN CK increased > 10× ULN Gastrointestinal disorders Muscle-related symptoms Muscle pain

Räber 2022	Europe Patients with acute myocardial infarction	Rosuvastatin 20 mg	151	58.6 ± 9.4	119 (78.3%)	52 weeks	ALT/AST increased > 3× ULN

Ran 2017	China Patients with acute coronary syndrome	Rosuvastatin 10 mg Rosuvastatin 20 mg	42 41	60.6 ± 6.7 60.5 ± 10.0	31 (73.8%) 30 (73.2%)	12 weeks	Adverse drug reactions ALT/AST increased > 3× ULN CK increased > 5× ULN Muscle pain Rhabdomyolysis

Cannon 2015	The United States and Europe Patients with acute coronary syndrome	Simvastatin 40 mg	9077	63.6 ± 9.8	6886 (75.9%)	2.5 years	ALT/AST increased > 3× ULN CK increased > 5× ULN Rhabdomyolysis Myopathy Gallbladder-related adverse events

Kyeong 2007	Korea Patients with acute coronary syndrome or acute ischemic stroke	Rosuvastatin 10 mg Atorvastatin 20 mg	77 79	63.5 ± 11.67 63.4 ± 10.88	41 (68.3%) 29 (50.9%)	40 weeks	ALT/AST increased > 3× ULN CK increased > 10× ULN Myalgia

Pierre 2007	Unclear Patients with transient ischemic attack (TIA) or stroke	Atorvastatin 80 mg	2365	63.0 ± 0.2	1427 (60.3%)	5 years	ALT/AST increased > 3× ULN CK increased > 10× ULN Myalgia Myopathy Rhabdomyolysis

Thompson 2004	Unclear Patients with acute coronary syndrome	Pravastatin 40 mg	1710	NA	1308 (76.4%)	30 days	ALT/AST increased > 3× ULN Myopathy

Lee 2020	Korea Patients with diabetes and hypercholesterolemia	Rosuvastatin 10 mg	68	56.9 ± 8.9	38 (55.9%)	8 weeks	ALT/AST increased > 3× ULN Myalgia

Thongtang 2020	Thailand Patients with Type 2 diabetes mellitus	Simvastatin 20 mg Atorvastatin 40 mg	76 74	58.9 ± 9.2 58.8 ± 8.6	18 (23.7%) 24 (32.4%)	12 weeks	Myalgia ALT/AST increased > 2× ULN

Bae 2019	Korea Patients with Type 2 diabetes and dyslipidemia	Rosuvastatin 20 mg	97	56.2 ± 9.2	49 (51.0%)	24 weeks	Adverse events Gastrointestinal disorder Myalgia

Knopp 2006	Fourteen countries (Australia, Canada, Switzerland, the United States, etc.) Patients with Type 2 diabetes and dyslipidemia	Atorvastatin 10 mg	1211	61.1 ± 8.1	796 (66%)	4 years	ALT/AST increased Myalgia Rhabdomyolysis

Gaudiani 2005	Unclear Patients with Type 2 diabetes mellitus	Simvastatin 40 mg	110	58.3	61 (55.5%)	24 weeks	Adverse drug reactions ALT/AST increased > 3× ULN CK increased > 10× ULN Myopathy

Berne 2005	Sweden Patients with Type 2 diabetic dyslipidemia	Rosuvastatin 40 mg Atorvastatin 80 mg	233 236	63.5 ± 8.8 65.0 ± 8.6	128 (44.8%) 136 (58.4%)	16 weeks	Adverse events ALT/AST increased > 3× ULN CK increased > 5× ULN Myalgia

## Data Availability

This study is a meta-analysis based exclusively on data extracted from previously published articles. All source studies are listed in the References section. The datasets generated during the current study (e.g., screening records and extracted numerical data) are available from the corresponding author upon reasonable request.

## Supporting Information

Supporting Information 1 Text S1: Search strategies of the PubMed, Embase, and Cochrane Library databases.

Supporting Information 2 Figure S1: Sensitivity analysis of studies about AE in hyperlipidemia patients.

Supporting Information 3 Figure S2: Sensitivity analysis of studies about ADRs in hyperlipidemia patients.

Supporting Information 4 Figure S3: Sensitivity analysis of studies about ALT/AST > 3× ULN in hyperlipidemia patients.

Supporting Information 5 Figure S4: Sensitivity analysis of studies about CK elevation in hyperlipidemia patients.

Supporting Information 6 Figure S5: Sensitivity analysis of studies about myalgia in hyperlipidemia patients.

Supporting Information 7 Figure S6: Sensitivity analysis of studies about gastrointestinal disorders in hyperlipidemia patients.

Supporting Information 8 Figure S7: Sensitivity analysis of studies about ALT/AST > 3× ULN in CHD patients.

Supporting Information 9 Figure S8: Sensitivity analysis of studies about myalgia in CHD patients.

Supporting Information 10 Figure S9: Sensitivity analysis of studies about myopathy in CHD patients.

Supporting Information 11 Figure S10: Sensitivity analysis of studies about rhabdomyolysis in CHD patients.

Supporting Information 12 Figure S11: Sensitivity analysis of studies about ALT/AST > 3× ULN in ACS or CIS patients.

Supporting Information 13 Figure S12: Sensitivity analysis of studies about myalgia in ACS or CIS patients.

Supporting Information 14 Figure S13: Sensitivity analysis of studies about ALT/AST > 3× ULN in diabetes patients.

Supporting Information 15 Figure S14: Sensitivity analysis of studies about myalgia in diabetes patients.
