# Correction to “A Meta‐Analysis of the Incidence of Adverse Reactions of Statins in Various Diseases”

**DOI:** 10.1155/cdr/9764793

**Published:** 2026-02-24

W. Li, D. Wang, C. Lin, et al., “A Meta‐Analysis of the Incidence of Adverse Reactions of Statins in Various Diseases,” *Cardiovascular Therapeutics* 2025, no. 1 (2025): 1–29, https://doi.org/10.1155/cdr/6684099.

In the article titled “A Meta‐Analysis of the Incidence of Adverse Reactions of Statins in Various Diseases,” incorrect funding details were included.

The correct funding statement should read:

This work was funded by the Fifth Batch of National Outstanding Traditional Chinese Medicine Clinical Talent Cultivation Program (National Administration of Traditional Chinese Medicine, Document No. [2022] 239) and the Guangxi First‐Class Discipline Construction Project (Gui Jiao Ke Yan [2022] No. 1).

We apologize for this error.

