# Supplementary material for: A Meta-Analysis of the Incidence of Adverse Reactions of Statins in Various Diseases
Source: Cardiovasc Ther. 2025 Jun 10;2025:6684099. doi: 10.1155/cdr/6684099 (PMC12173554; doi:10.1155/cdr/6684099)
Supplement: Supporting Information 1 — Text S1: Search strategies of the PubMed, Embase, and Cochrane Library databases. [file 6684099.f1.docx]

**Supplementary Text S1. Search strategies**

PubMed：

((statin[Title]) OR (rosuvastatin[Title]) OR (atorvastatin[Title]) OR (pitavastatin[Title]) OR (lovastatin[Title]) OR (fluvastatin[Title]) OR (pravastatin[Title]) OR (simvastatin[Title])) AND ((side effects[Title/Abstract]) OR (adverse effects[Title/Abstract]) OR (adverse reaction[Title/Abstract]) OR (adverse drugs reaction[Title/Abstract]) OR (adverse event[Title/Abstract])) AND (clinical trial[Publication Type])

Embase:

#1statin:ti OR rosuvastatin:ti OR atorvastatin:ti OR pitavastatin:ti OR lovastatin:ti OR fluvastatin:ti OR pravastatin:ti OR simvastatin:ti

#2'side effects':ab,ti OR 'adverse effects':ab,ti OR 'adverse reaction':ab,ti OR 'adverse drugs reaction':ab,ti OR 'adverse event':ab,ti

#3 'article'/it

#1AND #2 AND #3

Cochrane library：

#1 (statin):ti OR (rosuvastatin):ti OR (atorvastatin):ti OR (pitavastatin):ti OR (lovastatin):ti OR (Fluvastatin):ti

#2 (pravastatin):ti OR (simvastatin):ti

#3 (side effects):ab,ti,kw OR (adverse effects):ab,ti,kw OR (adverse reaction):ab,ti,kw OR (adverse drugs reaction):ab,ti,kw OR (adverse event):ab,ti,kw

#4 (randomize clinical study):ab,ti,kw

#5 (article):pt

#6(design):ti AND (rationale):ti

#1 OR #2 AND #3 AND #4 AND #5NOT#6
