# Supplementary figures and images for: A Meta-Analysis of the Incidence of Adverse Reactions of Statins in Various Diseases
Source: Cardiovasc Ther. 2025 Jun 10;2025:6684099. doi: 10.1155/cdr/6684099 (PMC12173554; doi:10.1155/cdr/6684099)

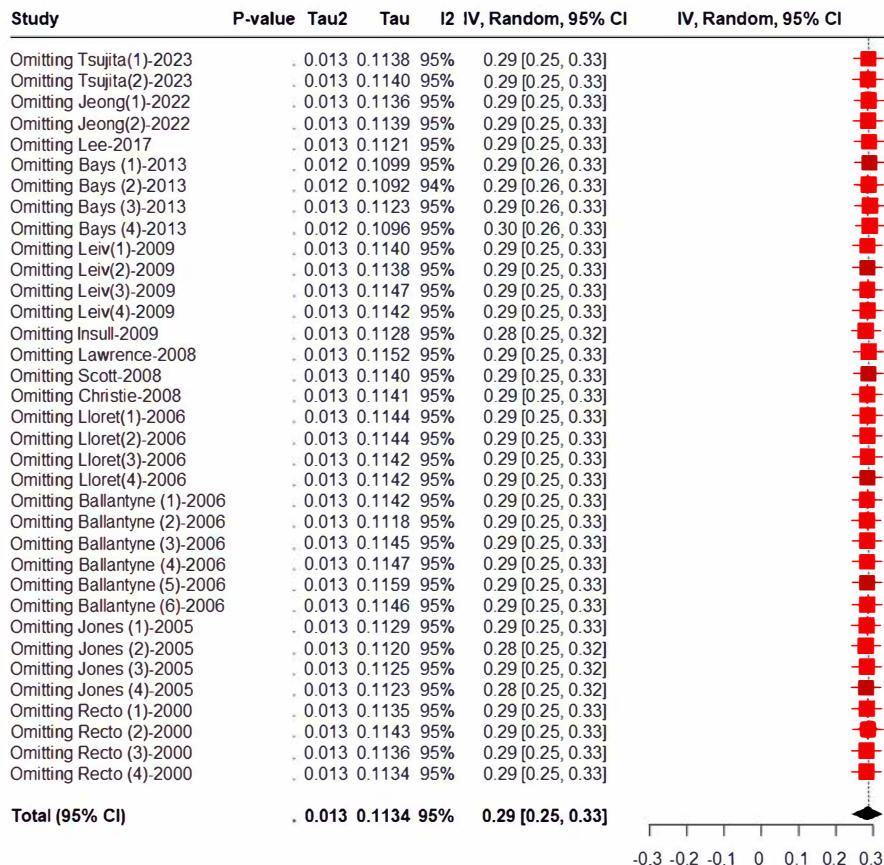

Figure S1 Sensitivity analysis of studies about AE in hyperlipidemia patients

Supplement: Supporting Information 2 — Figure S1: Sensitivity analysis of studies about AE in hyperlipidemia patients. [file 6684099.f2.pdf]

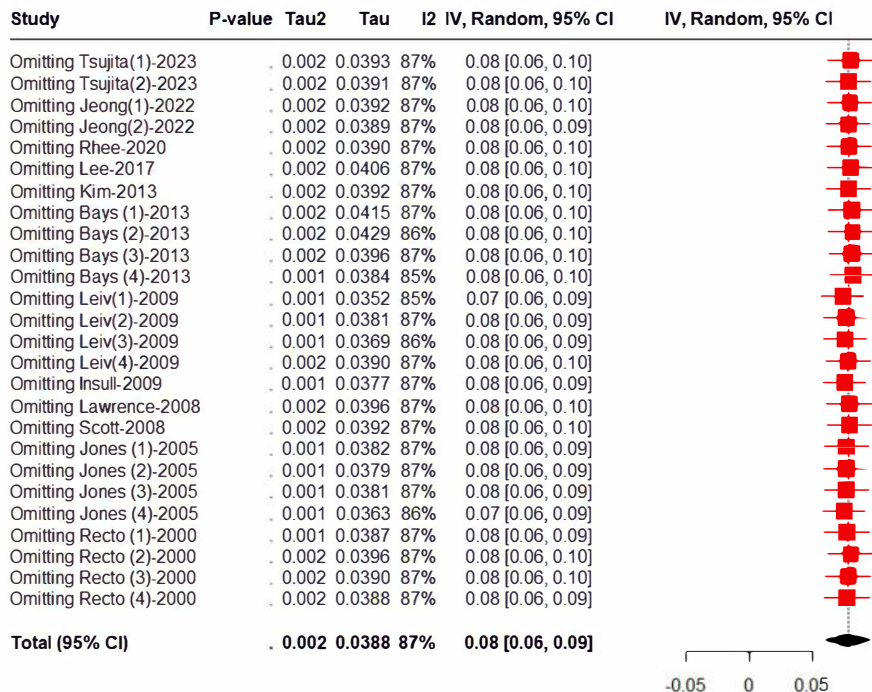

Figure S2 Sensitivity analysis of studies about ADRs in hyperlipidemia patients

Supplement: Supporting Information 3 — Figure S2: Sensitivity analysis of studies about ADRs in hyperlipidemia patients. [file 6684099.f3.pdf]

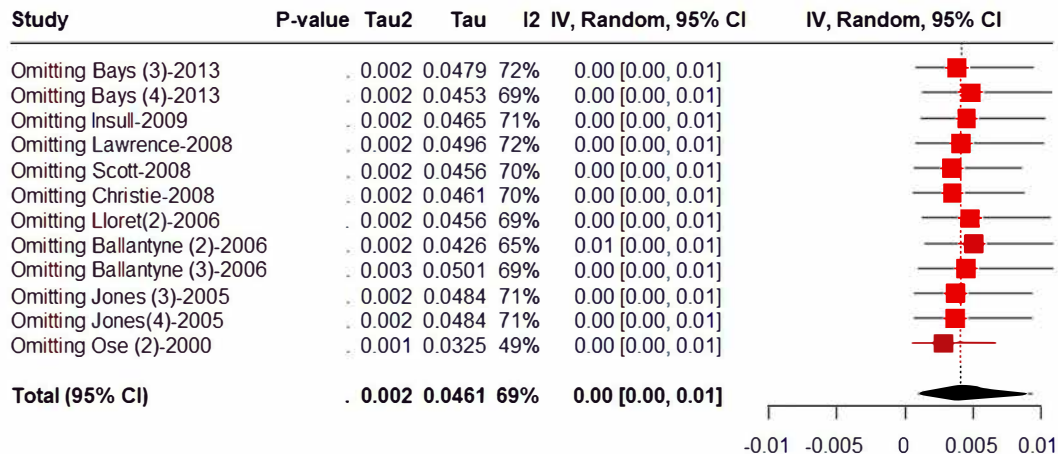

Figure S3 Sensitivity analysis of studies about ALT/AST>3x ULN in hyperlipidemia patients

Supplement: Supporting Information 4 — Figure S3: Sensitivity analysis of studies about ALT/AST > 3× ULN in hyperlipidemia patients. [file 6684099.f4.pdf]

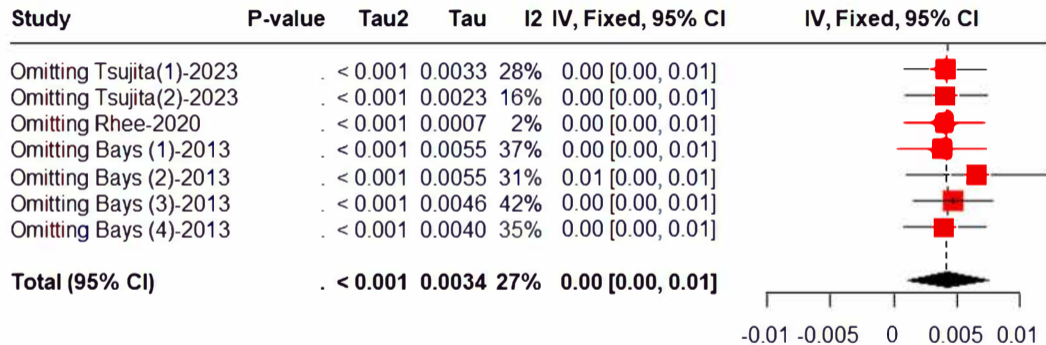

Figure S4 Sensitivity analysis of studies about CK elevation in hyperlipidemia patients

Supplement: Supporting Information 5 — Figure S4: Sensitivity analysis of studies about CK elevation in hyperlipidemia patients. [file 6684099.f5.pdf]

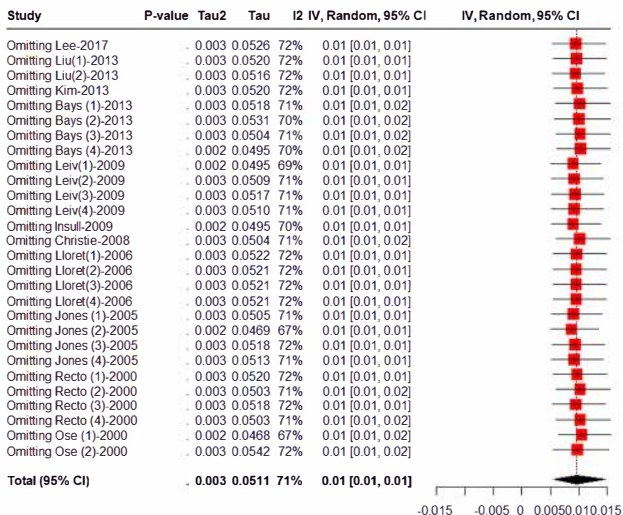

Figure S5 Sensitivity analysis of studies about myalgia in hyperlipidemia patients

Supplement: Supporting Information 6 — Figure S5: Sensitivity analysis of studies about myalgia in hyperlipidemia patients. [file 6684099.f6.pdf]

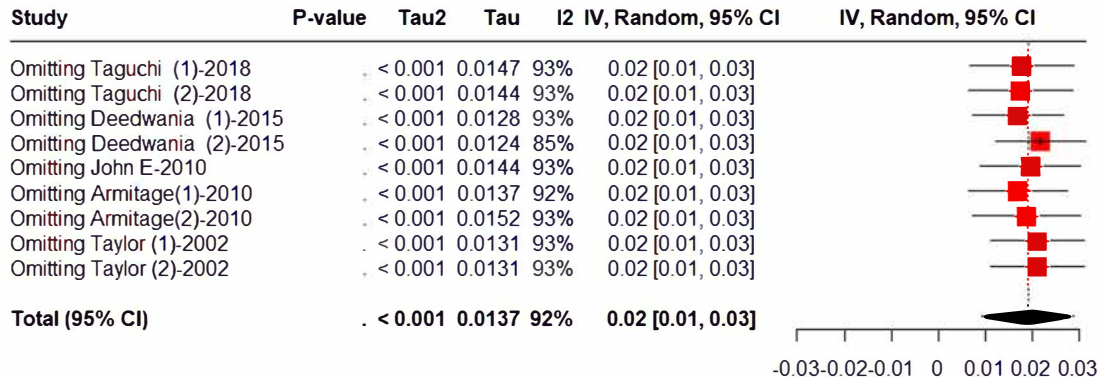

Figure S7 Sensitivity analysis of studies about ALT/AST >3x ULN in CHD patients

Supplement: Supporting Information 8 — Figure S7: Sensitivity analysis of studies about ALT/AST > 3× ULN in CHD patients. [file 6684099.f8.pdf]

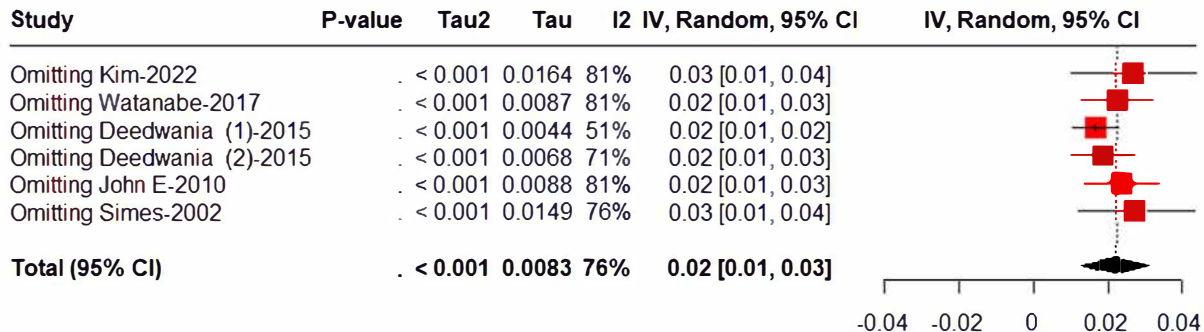

Figure S8 Sensitivity analysis of studies about myalgia in CHD patients

Supplement: Supporting Information 9 — Figure S8: Sensitivity analysis of studies about myalgia in CHD patients. [file 6684099.f9.pdf]

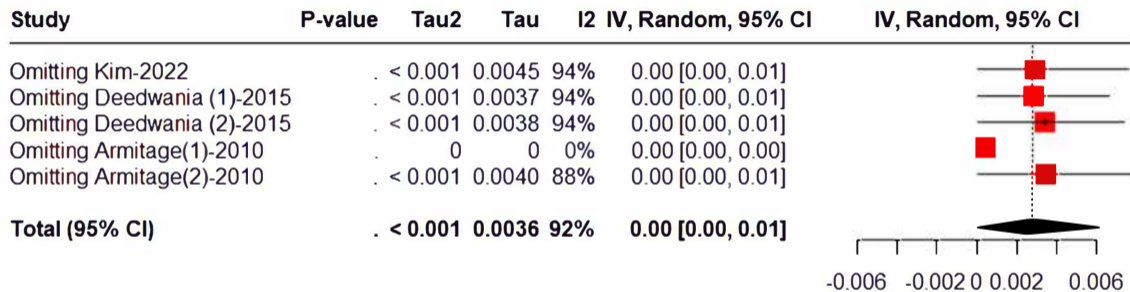

Figure S9 Sensitivity analysis of studies about myopathy in CHD patients

Supplement: Supporting Information 10 — Figure S9: Sensitivity analysis of studies about myopathy in CHD patients. [file 6684099.f10.pdf]

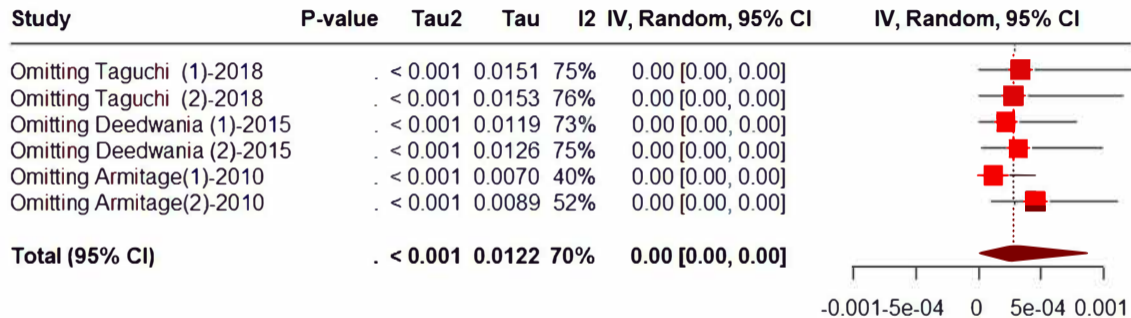

Figure S10 Sensitivity analysis of studies about rhabdomyolysis in CHD patients

Supplement: Supporting Information 11 — Figure S10: Sensitivity analysis of studies about rhabdomyolysis in CHD patients. [file 6684099.f11.pdf]

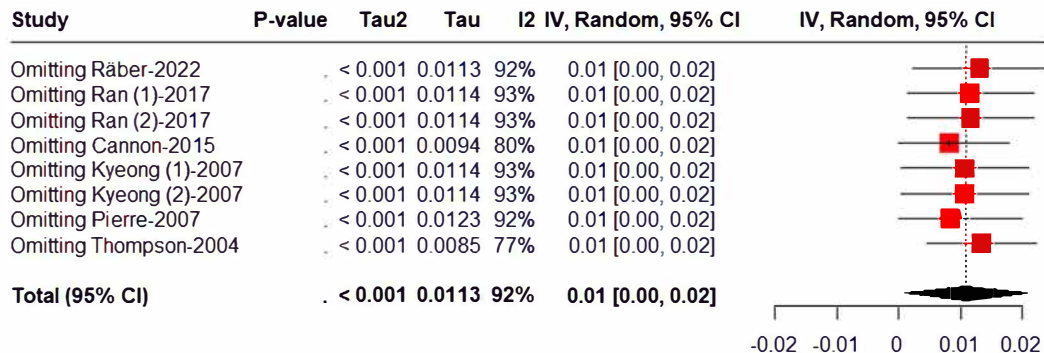

Figure S11 Sensitivity analysis of studies about ALT/AST >3x ULN in ACS or CIS patients

Supplement: Supporting Information 12 — Figure S11: Sensitivity analysis of studies about ALT/AST > 3× ULN in ACS or CIS patients. [file 6684099.f12.pdf]

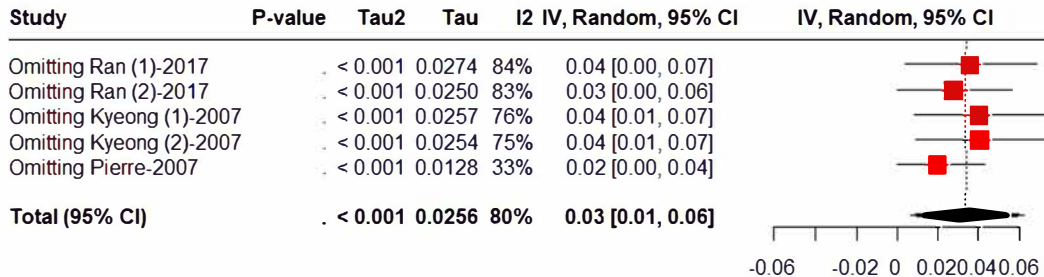

Figure S12 Sensitivity analysis of stt1dies about myalgia in ACS or CIS patients

Supplement: Supporting Information 13 — Figure S12: Sensitivity analysis of studies about myalgia in ACS or CIS patients. [file 6684099.f13.pdf]

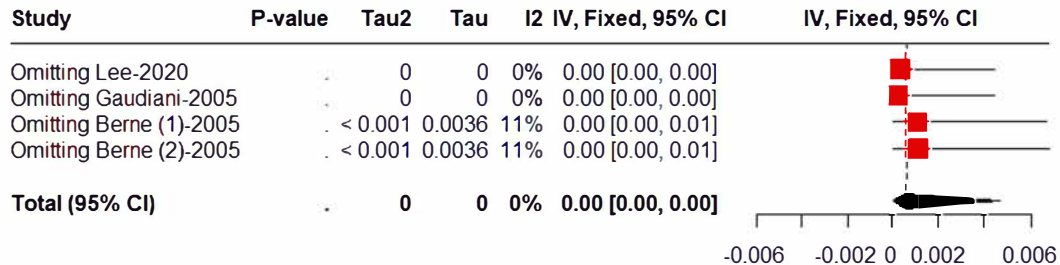

Figure S13 Sensitivity analysis of studies about ALT/AST >3x ULN in diabetes patients

Supplement: Supporting Information 14 — Figure S13: Sensitivity analysis of studies about ALT/AST > 3× ULN in diabetes patients. [file 6684099.f14.pdf]

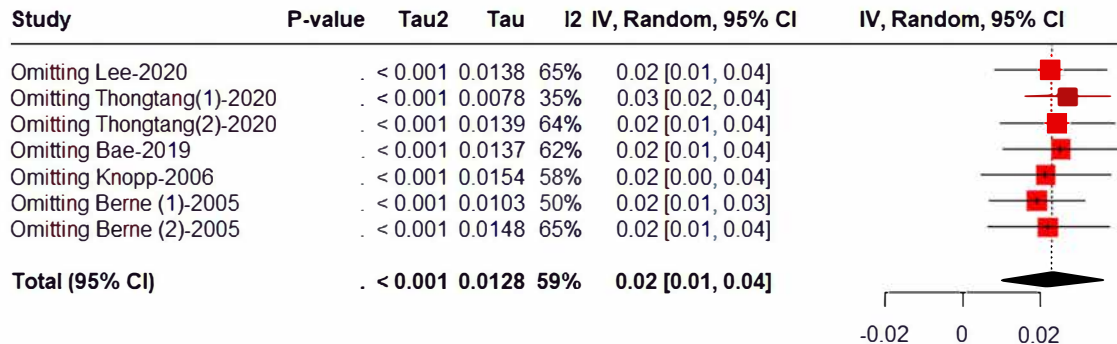

Figure S14 Sensitivity analysis of studies about myalgia in diabetes patients

Supplement: Supporting Information 15 — Figure S14: Sensitivity analysis of studies about myalgia in diabetes patients. [file 6684099.f15.pdf]
