# Supplementary material for: A Meta-Analysis of the Incidence of Adverse Reactions of Statins in Various Diseases
Source: Cardiovasc Ther. 2025 Jun 10;2025:6684099. doi: 10.1155/cdr/6684099 (PMC12173554; doi:10.1155/cdr/6684099)
Supplement: Supporting Information 7 — Figure S6: Sensitivity analysis of studies about gastrointestinal disorders in hyperlipidemia patients. [file 6684099.f7.pdf]

Study	P-value	Tau2	Tau	I2	IV, Random, 95% CI	IV, Random, 95% CI
Omitting Jeong(1)-2022	.	< 0.001	0.0099	56%	0.02 [0.01, 0.03]	
Omitting Jeong(2)-2022	.	< 0.001	0.0105	58%	0.02 [0.01, 0.04]	
Omitting Kim-2013	.	< 0.001	0.0088	50%	0.02 [0.01, 0.03]	
Omitting Bays (1)-2013	.	< 0.001	0.0114	57%	0.02 [0.01, 0.04]	
Omitting Bays (2)-2013	.	< 0.001	0.0130	58%	0.03 [0.01, 0.04]	
Omitting Bays (3)-2013	.	< 0.001	0.0097	50%	0.03 [0.02, 0.04]	
Omitting Bays (4)-2013	.	< 0.001	0.0099	49%	0.03 [0.02, 0.04]	
Omitting Lawrence-2008	.	< 0.001	0.0065	34%	0.02 [0.01, 0.03]	
Omitting Scott-2008	.	< 0.001	0.0093	53%	0.02 [0.01, 0.03]	
**Total (95% CI)**	**.**	**< 0.001**	**0.0097**	**52%**	**0.02 [0.01, 0.03]**	

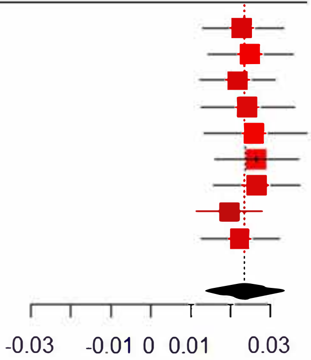

Figure S6 Sensitivity analysis of studies about gastrointestinal disorders in hyperlipidemia patients
